# Environmental optima for an ecosystem engineer: a multidisciplinary trait-based approach

**DOI:** 10.1038/s41598-021-02351-7

**Published:** 2021-11-26

**Authors:** Amelia Curd, Aurélien Boyé, Céline Cordier, Fabrice Pernet, Louise B. Firth, Laura E. Bush, Andrew J. Davies, Fernando P. Lima, Claudia Meneghesso, Claudie Quéré, Rui Seabra, Mickaël Vasquez, Stanislas F. Dubois

**Affiliations:** 1 IFREMER, Centre de Bretagne, DYNECO LEBCO, 29280 Plouzané, France; 2 LEMAR CNRS/UBO/IRD/Ifremer, ZI pointe du diable, CS 10070, 29280 Plouzané, France; 3 School of Biological and Marine Sciences, University of Plymouth, Drake Circus, Plymouth, PL4 8AA UK; 4 FUGRO GB Marine Limited, Heriot-Watt University, 1-9 The Curve, 32 Research Avenue North, Edinburgh, EH14 4AP UK; 5 Department of Biological Sciences, University of Rhode Island, Kingston, RI 02881 USA; 6 CIBIO-InBIO, Centro de Investigação em Biodiversidade e Recursos Genéticos, Universidade do Porto, Campus Agrário de Vairão, 4485-661 Vairão, Portugal; 7 Departamento de Biologia, Faculdade de Ciências da Universidade do Porto, R. Campo Alegre, s/n, 4169-007 Porto, Portugal

**Keywords:** Biooceanography, Ecophysiology, Macroecology, Animal physiology

## Abstract

A complex interplay of biotic and abiotic factors underpins the distribution of species and operates across different levels of biological organization and life history stages. Understanding ecosystem engineer reproductive traits is critical for comprehending and managing the biodiversity-rich habitats they create. Little is known about how the reproduction of the reef-forming worm, *Sabellaria alveolata*, varies across environmental gradients. By integrating broad-scale environmental data with in-situ physiological data in the form of biochemical traits, we identified and ranked the drivers of intraspecific reproductive trait variability (ITV). ITV was highest in locations with variable environmental conditions, subjected to fluctuating temperature and hydrodynamic conditions. Our trait selection pointed to poleward sites being the most physiologically stressful, with low numbers of irregularly shaped eggs suggesting potentially reduced reproductive success. Centre-range individuals allocated the most energy to reproduction, with the highest number of intermediate-sized eggs, whilst equatorward sites were the least physiologically stressful, thus confirming the warm-adapted nature of our model organism. Variation in total egg diameter and relative fecundity were influenced by a combination of environmental conditions, which changed depending on the trait and sampling period. An integrated approach involving biochemical and reproductive traits is essential for understanding macro-scale patterns in the face of anthropogenic-induced climate change across environmental and latitudinal gradients.

## Introduction

Understanding how patterns of species distribution and abundance will manifest in a changing world remains a core goal in ecology. The factors shaping a species’ distribution should be reflected in the geographic variation of their life-history traits^1^. In the marine environment, the roles of temperature and repopulation (defined as the combination of reproduction and recruitment), in setting latitudinal distribution limits have long been known^2^. Range limits can be set by either abiotic or biotic factors. Among the latter, reproductive success is acknowledged to be a critical factor in determining a species range edge^2,3^.

Spatial patterns in reproductive traits have several theoretical explanations but have been rarely tested empirically across species’ entire geographic ranges. Offspring size is perhaps the most studied trait^4^. In the marine environment, a strong relationship between offspring size, development mode and latitude has been long-established^5,6^: for many taxa, eggs and feeding larvae are small at lower latitudes, whereas higher-latitude taxa tend to have larger eggs and non-feeding larvae. However, the mechanisms mediating latitudinal variation in reproductive traits are unclear. Average conditions, but also the seasonality and predictability of conditions, differ dramatically across latitude, all of which play a part in selection on, and shape spatial patterns in offspring size^7^. The causes and consequences of within-species and within-clutch variation in offspring size are even less well understood.

Variation within a species (intraspecific trait variability, ITV) can have as great an effect on ecological processes as variation among species^8^. Traits can be described as any morphological, physiological, phenological or behavioural feature measurable at the individual level^9^. Species may be able to adjust to a wider range of biotic and abiotic conditions as a consequence of greater ITV and therefore, have greater niche breadth^10^. ITV may vary due to heritable differences between individuals, non-heritable genetic effects (i.e. epistasis or dominance) or because of phenotypic plasticity across varying environmental conditions^11^. While ITV across large geographic areas can often be related to environmental gradients^12^, it remains poorly understood whether it is associated with environmental heterogeneity at a local scale^13^.

The number and size of offspring are arguably the two most important and variable life-history traits for population persistence^4^. There are a proliferation of studies on the intuitive, though surprisingly rarely demonstrated^13^, offspring size-number trade-off, first formally modelled in the 1970s^14^. Aside from offspring size and number, little is known about the spatial patterns in other reproductive traits such as gamete shape, quality, and maternal physiological condition, particularly across a species range. Gamete traits, and conditions of sperm availability, lead to different patterns of average and variance in reproductive success^15^.

Abiotic environmental changes have direct impacts on traits, which govern how species respond to different environmental filters^16^. For sessile organisms, where adults are unable to escape unfavorable conditions, reproductive traits determine the viability of local adult populations, which in turn can have community-level consequences if those species are ecosystem engineers^17^. These alterations to proximal organismal-level processes can scale-up to changes in community structure, which ultimately lead to emergent ecological responses such as biogeographic shifts^18^.

ITV is not always beneficial. While it has long been known that ITV reflects the ability of a species to exist in a more diverse range of environments, greater ITV within a population means that more individuals have a trait value further from the optimum, thus lowering the overall population mean fitness. Outside of the plant ecology field, the patterns and drivers of ITV, and their link to species resilience, remain for the most part unknown^19^. Given ITV in egg number and size in female marine invertebrates is common^20^, such species are ideal models for determining the effects of environmental stressors on ITV and for understanding potential adaptation strategies in a changing world. Studies have investigated the effects of temperature, food availability, or other physical factors on the physiology of marine animals and have led to the development of biochemical indicators of metabolic condition, and physiological stress^21^. Some biochemical indicators are particularly informative of the organismal stress generated by gametogenesis and egg production^22^. Few studies in marine ecology have examined how reproductive traits and maternal physiological condition vary spatially at the scale of species’ ranges^1,23^, and to our knowledge, none have quantified the amount of ITV attributable to local *vs*. entire geographic range scale environmental variables.

### Study system

Intertidal invertebrates live on the edge of two worlds and are thus exposed to environmental challenges posed by both the terrestrial and marine realms^24^. Their unique position means they are doubly exposed to climate stressors, with combined effects of rising and fluctuating air and seawater temperatures (and indeed other factors) having a large impact on many natural assemblages^25^. The reef-forming worm, *Sabellaria alveolata* L. is a broadly distributed intertidal species that engineers a unique high-biodiversity habitat^26–28^ by cementing together coarse sand grains and shell fragments into tubes. Dense aggregations of these tubes form biogenic reefs^29^, which are afforded statutory protection by the European Union’s Habitat Directive (Council Directive 94/43/EEC). *S. alveolata* is distributed in the Mediterranean from Scotland to Morocco and, as a warm-adapted species, is known to be negatively affected by cold weather spells^30–32^. As environmental impacts on ecosystem engineers may result in cascading effects on ecosystem structure and functioning^33^, we chose to study this species as a pragmatic first step towards gaining insight into the ecological impacts of reproductive responses to environmental stressors.

This study aimed to explore both the geographic and within-site variation in an ecosystem engineers’ response traits. Using biochemical and microscopic imagery analyses, we examined the phenotypic responses in the reproductive traits and maternal physiological condition of intertidal *S. alveolata* to environmental variables, over two seasons (winter and summer), across ten sites, encompassing almost its entire latitudinal range across Atlantic Europe. We explored patterns in several reproductive traits before focusing on egg size and number, and their environmental and physiological drivers. We investigated the relationship between maternal physiological condition and reproductive traits, and whether these varied with respect to environmental parameters across a latitudinal gradient. We quantified the proportion of variability associated with site-scale environmental and biochemical predictors. Finally, we tested the hypothesis that ITV would be highest in the locations where *S. alveolata* is subjected to greater environmental stress, such as extreme or fluctuating abiotic conditions.

## Results

### Latitudinal structures in reproductive and biochemical traits

**Figure 1:**
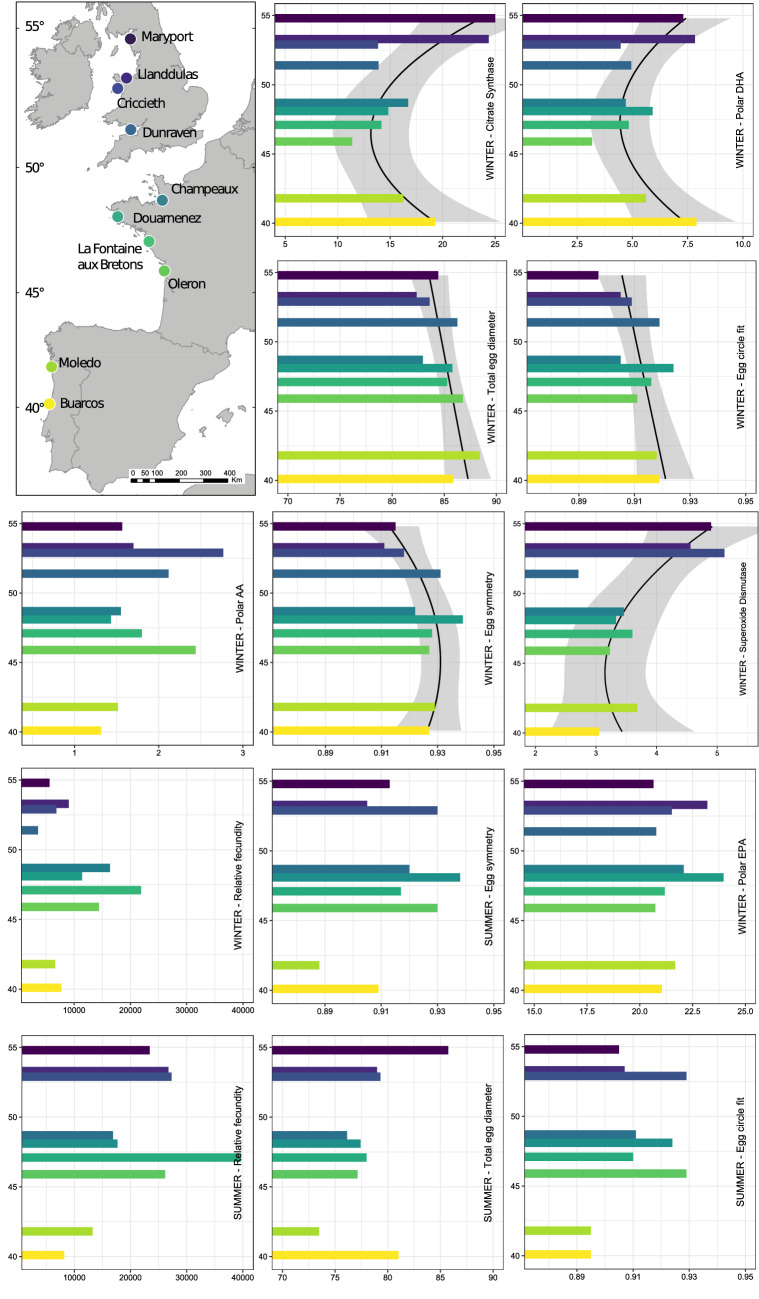
Sampling sites and barplots of among-female means in key reproductive and biochemical *S. alveolata* traits vs. latitude. Latitude was treated as the independent variable and the axes were then flipped for presentation purposes. When the relationship with either linear (equator- or pole-ramped) or quadratic latitude (abundant edge or abundant center) was significant (*p*-value < 0.05), regressions with standard errors were plotted. x-axis units and abbreviations are as follows: total egg diameter = µm; relative fecundity = the number of eggs divided by the opercular crown diameter of the adult female worm; egg circle fit = [0–1], index, where 1 is a perfect circle; egg symmetry = [0–1], index, where 1 is perfect symmetry; citrate synthase = micro Units of protein (mU mg^−1^); superoxide dismutase = Units of protein (U mg^−1^); Polar DHA = docosahexaenoic acid in the polar lipid fraction = % of total phospholipids. Polar AA = arachidonic acid in the polar lipid fraction = % of total phospholipids. Polar EHA = eicosapentanoic in the polar lipid fraction = % of total phospholipids. Sampling was carried out either in summer 2017 or winter 2018.

Trait latitudinal distributions can follow one of four patterns^34^: they can either be ramped towards the pole or equator, or have an abundant centre or edge shape (see Supp. Table S1). Our results were variable among traits and sampling times, with significant distribution patterns being found in six out of the 13 traits examined. Citrate synthase (CS), polar lipid docosahexaenoic acid (DHA) and superoxide dismutase followed an abundant edge pattern, winter egg symmetry followed an abundant centre pattern, whilst winter total egg diameter and egg circle fit were pole-ramped (Fig. 1). Relative fecundity during both seasons, together with summer egg symmetry, total egg diameter, egg circle fit and relative fecundity, polar lipid eicosapentaenoic acid (EPA) and polar lipid arachidonic acid (AA) showed no relationship with latitude.

### Relationship between reproductive and biochemical traits

**Figure 2:**
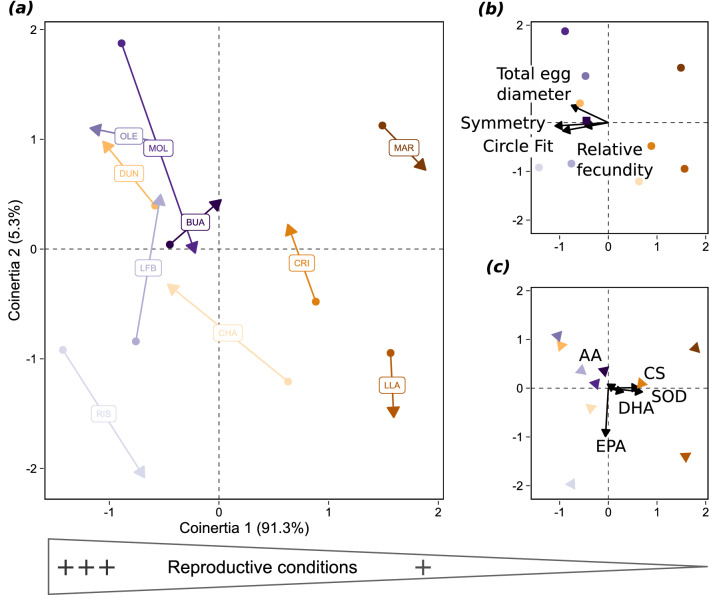
Graphical results of the co-inertia analysis of the reproductive and biochemical variables for winter 2018. The left-hand plot (**a**) (normed site-scores) shows the position of the sites on the co-inertia axes using the reproductive (origins of the arrows) and biochemical (arrowheads) co-inertia weights. The shorter the arrows, the better the match between the two projections. The right-hand pair of plots shows the contribution of the two groups of variables to the canonical space (reproductive traits (**b**) on the top; biochemical variables on the bottom (**c**)). Vectors pointing in the same direction are correlated and longer vectors contribute more to the structure. Site name abbreviations are as follows: Maryport MAR, Llanddulas LLA, Criccieth CRI, Champeaux CHA, Douarnenez, plage du Ris RIS, La Fontaine aux Bretons LFB, Oléron OLE, Moledo MOL, Buarcos BUA. Biochemical abbreviations as in Fig. 1.

Although the relationship between reproductive trait and mean biochemical composition variation is moderate (RV coefficient of 0.38), these two sets of variables share a common structure, represented along the first co-inertia axis, which accounts for 91.3% of the covariance (Fig. 2). The covariance between the reproductive and biochemical datasets clearly separated the three poleward sites (Maryport MAR, Llanddulas LLA and Criccieth CRI) from the remaining seven sites (Fig. 2). This is due to their high levels of metabolic activity (CS and SOD) and polar lipid DHA levels, as shown by the vectors of these three variables pointing towards the sites positions, in combination with eggs with low symmetry and circle fit indices, as shown by the latter two vectors pointing in the opposite direction along the first axis. Together with the equatorward most site (Buarcos BUA), these four sites had the highest agreement between the two sets of variables, as depicted by the shorter vectors in Fig. 2a. Conversely, the sites showing the least agreement between biochemical and reproductive variables (the longer vectors in Fig. 2a) are located in the centre-equatorward portion of the range (Champeaux CHA, Douarnenez RIS, la Fontaine aux Bretons LFB, Moledo MOL). Sites with high egg symmetry and best circle fit in winter 2018 had low levels of metabolic enzyme activity (SOD, CS), were found in the centre to equatorward part of the range (Figs. 1, 2; illustrated by the opposite vectors in Fig. 2b,c and the positioning of the centre-range sites from Douarnenez RIS to Buarcos BUA on the left of Fig. 2a).

### Drivers of ITV and environmental heterogeneity

**Figure 3:**
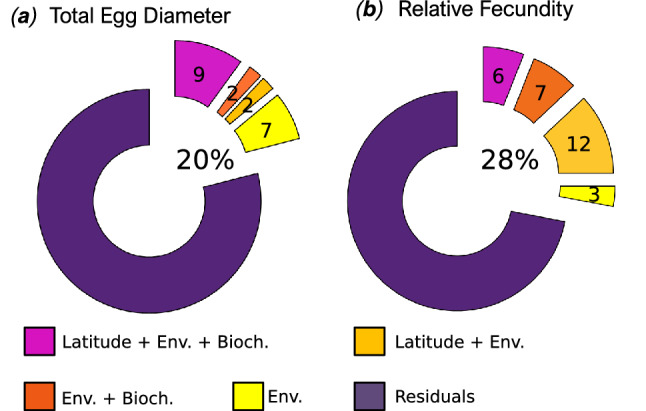
RDA variance partitioning of among-female egg size and relative fecundity during winter 2018. Numbers indicate R_adj_^2^ values. Only the non-null fractions and residuals are represented. The four non-null fractions were: (**a**) environmental predictors alone (Env.), (**b**) the shared effect of environmental predictors with latitude (Latitude + Env.), (**c**) the shared effect of biochemical and environmental predictors (Env. + Bioch.) and (**d**) the shared effect of biochemical and environmental variables together with latitude (Latitude + Env. + Bioch). Latitude is linear for total egg diameter, and quadratic for relative fecundity. Environmental variable selection for each trait was based on the RDA analysis presented in Fig. 4.

The majority of variation occurring in total egg diameter and relative fecundity in winter was not attributed to our selected environmental, biochemical and spatial predictors, as shown by the large fraction of residuals in our variance partitioning analysis (Fig. 3). Our selected environmental, biochemical and spatial predictors captured 20% of ITV in total egg diameter and 28% of ITV in relative fecundity. For total egg diameter, 11% of the ITV explained by environmental variables was linearly structured by latitude t (Fig. 3a), with 9% being shared with biochemical variables. For relative fecundity, 18% of the ITV explained by environmental variables was spatially structured (shared here with the quadratic polynomial of latitude), with 6% being shared with biochemical variables (Fig. 3b).

**Figure 4:**
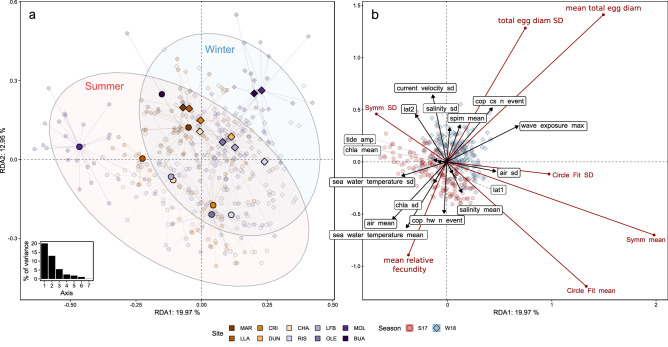
Redundancy analysis (RDA) of selected centered and standardized within-clutch reproductive traits. Reproductive traits are displayed as red vectors in the right-hand (**b**); (Scaling 2) constrained by selected standardized environmental variables (displayed as black vectors (**b**); Scaling 2). The left-hand (**a**) shows the centroid scores for each sampling site and season, linked to each individual (Scaling 1). 95% confidence dispersion ellipses are represented for the seasons. In both panels, summer scores are displayed by circles, winter scores as diamonds and colors correspond to the sampling sites. Axes values represent the % of explained variance per axis, the two axes representing altogether 33% of the total variance. The inner figure of (**a**) corresponds to the eigenvalues of the first 7 RDA axes. (**b**) Environmental variable abbreviations are as follows: ‘spim mean’ = mean suspended particulate inorganic matter; ‘lat1’ = linear latitude; ‘lat2’ = quadratic latitude; ‘chla’ = chlorophyll-*a*; ‘tide amp’ = mean tidal amplitude; ‘cop cs n event’ = number of cold spells in seawater over 30 days; ‘cop hw n event’ = number of heatwaves in seawater over 30 days; ‘air mean’ = air temperature mean. All ‘sd’ suffixes represent the variation (standard deviation) of different variables. Site abbreviations as for Fig. 2.

When examining all reproductive traits and both sampling seasons, winter and summer separated out along the first axis of the redundancy analysis plot (Fig. 4a,b). Our set of environmental variables explained 33% of their variation and co-variation. Winter conditions, namely higher maximum wave exposure, number of cold spells, suspended particulate inorganic matter concentrations and stronger variance in current velocities, were linked to higher mean and standard deviation in total egg diameter and lower relative fecundity. Conversely, summer conditions, characterized by higher air and seawater temperatures, heat wave events and variable chlorophyll-*a* were associated with smaller, more uniformly-sized eggs, but higher relative fecundity (Fig. 4b). Egg best circle fit and symmetry exhibited a weaker correlation with environmental variables, although higher variance in air temperature and mean salinity, as well as lower variance in current velocity, appear to promote more symmetrical and circular eggs (Fig. 4b).

## Discussion

To the best our best knowledge, this is the first study which evaluates variability in reproductive traits while relating them to biochemical indicators of maternal physiological condition across a species geographical range. Despite considerable environmental heterogeneity of the Atlantic coast of Europe, six out of 13 traits displayed a relationship with latitude. Based on the covariance in biochemical and reproductive traits in winter, our three poleward sites were clearly separated from the remaining seven as ‘physiologically stressful’ sites showing low numbers of irregularly shaped eggs. Overall, biochemical traits indicated that the best physiological conditions occurred towards the equator, thus corroborating the warm-adapted nature of *S. alveolata*. The individuals investing the most in reproduction, with the highest relative fecundity by far (yet not the smallest eggs), were from the centre of the range. ITV quantification through our variance component models demonstrated that the majority of variation occurred at a within-site scale, which is finer than the relatively coarse resolution of our remotely-sensed environmental data. The fraction of variability explained by environmental data, however, suggests a complex interplay of environmental conditions associated with reproductive trait variation, specifically variability in temperature (cold spells and heatwaves), variability in current velocity, and peaks in primary production. Our data suggest that there are both multiple phenological drivers influencing maternal physiology and performance, and multiple proximate cues shaping gamete production.

Our data suggest that environmental conditions in the three most poleward sites were physiologically more stressful for *S. alveolata* than those encountered in the centre or equatorward sites. These sites display the least circular eggs together with the highest levels of SOD and AA. SOD reflects oxidative stress levels and has been linked to thermal stress in marine bivalves^35^, yet in our study SOD rose with increasing latitude and decreasing temperature. AA is a precursor of eicosanoids, known to be associated in other invertebrates with stressful or energetically expensive situations such as gametogenesis, spawning or immune function stimulation^36,37^. Ordinarily, AA increases with increasing seawater temperature, although the underlying reasons are not currently understood^38,39^. Collectively, these biochemical and reproductive traits provide evidence that environmental conditions towards the poleward limit of this warm-adapted species are physiologically stressful.

We found reproductive investment in *S. alveolata* to be greatest in the centre of its range. The four centre sites displayed the most symmetrical eggs, the highest relative fecundity, and the lowest levels of CS and polar lipid DHA. Both CS and DHA are highly correlated (R = 0.76) and related to aerobic metabolic rate^40^. Given the high relative fecundity displayed in the four centre sites, an active aerobic metabolism (as displayed by depleted CS and DHA levels) in *S. alveolata* could indicate energy allocation towards reproduction*.* This is supported by a study on tropical scallops, where CS levels were found to decrease during gonadal maturation and after spawning^41^.

Egg size is typically the functional trait by which mothers buffer their young from harsh environmental conditions. Much work has been done on analysing the influence of average isolated environmental variables on egg size, particularly temperature^42,43^. Our results suggest that higher variability in environmental conditions leads to greater ITV in reproductive traits. Summer variation in chlorophyll-*a* production, and episodic cold spells or heat waves across both sampling periods, led to greater variability in total egg diameter. This conforms with the theory that mothers increase the level of within-clutch variation in offspring size when environments are less predictable^44^. As chlorophyll-*a* production follows phytoplankton blooms, variable spikes in its concentration are an indicator of productivity^45^. Greater variation in phytoplankton bloom phenology is predicted under future global warming scenarios^46^ which may have repercussions for adult *S. alveolata* and other suspension-feeders, as well as their planktotrophic larvae.

Despite large uncertainty in climate projections, wind/wave forecasts are predicted to change significantly^47^, and an increased frequency and severity in heat waves is already occurring^48^. Variation in air temperature and maximal wave exposure positively influenced variability in *S. alveolata* egg circularity, and surface current variation was positively correlated with variability in egg symmetry. Several studies found sabellariids to have facultative breeding peaks following major storms and wave-related disturbances^49,50^, a phenomenon also observed in other intertidal species^51^. Additionally, the number of seawater temperature cold spell events over the preceding 30 days was positively correlated with both the diameter and variability in total egg size in winter. Whilst the majority of studies assessing the impacts of extreme weather events focus on heat stress (e.g.^39,52,53^), relatively little work has been carried out on cold stress (but see^54,74^).

Our quantification of the spatial variation in total egg diameter and relative fecundity revealed that the majority of variance occurred within-rather than among sites, except for summer total egg diameter. This within-site heterogeneity suggests many local habitat factors operate to a greater effect than latitudinal factors^3^ and cannot therefore be elucidated by site-scale predictors. Nonetheless, our selected broad-scale environmental and biochemical predictors captured most of the explainable inter-site variability in total egg diameter, although they performed more poorly for relative fecundity. Only 28% of ITV (out of 41% of inter-site variance on average) was captured by our site-scale predictors for relative fecundity. This means that either our data has missed some of its potential drivers of variation, or that our environmental data sources did not adequately capture the local environment responsible for phenotypic plasticity^24^. Whilst they are the result of daily model outputs and can be considered to be at a high temporal resolution, their spatial coarseness does not always reflect local conditions^55,56^.

Marine invertebrate populations have traditionally been viewed as being strongly structured by the quantity of offspring; however, offspring quality has an equally important effect^57^. The widespread use of averaged values for the functional traits of a species sampled in a given location signifies an implicit assumption that ITV is negligible compared to interspecific trait variation^11^. While often regarded as ‘noise’, ITV is key to the fundamental processes of natural selection and speciation, and should therefore be examined in trait-based research in both species-specific and community ecology^58^. In both sampling periods, total egg diameter variability was lowest and mean relative fecundity peaked in the centre of the range. If conditions during larval development and maturation are favourable, many small offspring are the best reproductive strategy, however if conditions are unexpectedly harsh, the whole clutch can be lost^59^.

We make the case for using the symmetry and circularity of *S. alveolata* egg vitelline membranes as indicators of gamete quality, although we acknowledge that the link with a performance metric such as fertilization success still needs to be demonstrated. With the advent of high-speed imaging flow cytometers, it is now possible to analyse dozens of physical gamete parameters and link these to their viability, enabling the use of many more reproductive traits. Very few studies on the biochemical quality of marine invertebrate gametes exist, and those that do, typically involve echinoids or mollusks (see Ref.^57^ for review). The fact that we now have the capability to perform near-exhaustive analyses of physiological markers and reproductive traits provides a more nuanced background against which ecological theories could be tested and challenged.

Macrophysiological studies, where traits are compared between individuals separated across large geographic scales, can advance our understanding of complex macro-scale phenomena such as biological invasions and species responses to global climate change^60^. However, the general conclusions reached by this approach can be influenced by a number of confounding variables; phylogeny is one such constraint^61^. More data on intraspecific trends in reproductive traits along latitudinal gradients would be useful, particularly studies that separate genetic from environmental influences^42^. It will also be important to determine the heritability of changes to reproductive traits induced by exposure to climate change stressors^20^, as transgenerational plasticity enables evolutionary responses to selection. Broad-scale empirical field studies of biogenic habitat-forming species must continue, for they allow us to detect general patterns over large scales, which in turn are vital for informing regional environmental policy^24,62^. For ecosystem engineers, small environmentally induced shifts in metabolic activity, brought on by non-lethal but highly stressful conditions such as those experienced during cold spells, may lead to disproportionately large impacts on biodiversity and ecosystem functioning through changes in reproduction, growth and survival^63^.

This study adds to the mounting evidence that the poleward edge populations of *S. alveolata* are vulnerable. Not only is this where individuals are in the poorest physiological condition, but it is also where the greatest genetic diversity^64^, and population instability^31^, are found. Taken together with the long-term stability of the poleward-most range edges both in Ireland^32^ and in Britain^65^, it is likely that *S. alveolata* will not be able to advance beyond these edges, even under future climate change scenarios. Only by combining fine-scale data that captures local conditions and proximate physiological responses with regional scale environmental information can we gain an understanding of emergent ecological and biogeographic responses. A better grasp of which environmental variables play a role in the physiological response of intertidal organisms will come with further integration of controlled experimental studies^52^. Nevertheless by studying the range-wide variability in novel reproductive and biochemical traits in a foundation species, together with characterising the abiotic environment, we bring new perspective to the relationship between species and habitats.

## Methods

We measured reproductive traits and biochemical indicators of physiological condition in individuals from ten populations of *S. alveolata*, distributed from northwest England to central Portugal (14.5° latitude, Fig. 1). The northernmost site is very close to its poleward range limit and the southernmost site is relatively close to the known equatorward range limit in Morocco^66^. We focused on females, which are more sensitive to environmental stressors than males due to the higher energetic cost in producing eggs compared to sperm^20^, although the latter are also sensitive to abiotic factors^67^.

### Animal collection

Ten sites were sampled during winter 2018, and nine sites (Dunraven was omitted) during summer 2017, all during spring tides (see Supplementary Table S2 online). *S. alveolata* is a continuous broadcast trickle spawner, meaning that a varying proportion of ripe worms can always be found within a reef^68^. Small clumps of tubes were broken off from mid-tide level reefs and 60 ripe female individuals were removed from their tubes. Ripe females are externally recognisable as the abdominal segments are swollen and appear pink^69^. In order to extract the eggs, half of the individuals were immediately placed in Falcon tubes containing 9 ml filtered seawater, agitated and left for 1 h, before adding 1 ml buffered 39% formalin solution (i.e. made up to 10 ml of 4.0% formaldehyde)^65^. For biochemical analyses, the other half of the individuals had their rigid opercular crowns separated from their abdominal and posterior segments with a sharp blade. Opercular crowns were preserved in individual Eppendorf tubes containing 39% formalin solution for subsequent size measurements, while the corresponding abdomen and posterior segments were placed in individual cryotubes and immediately flash frozen in liquid nitrogen in situ*.* The opercular crown diameter measurement of all 60 worms (the only hard morphological structure) was used as a recognised proxy for size^70^. The cryotubes were then stored at − 80 °C until laboratory biochemical analyses.

### Environmental variables

For each of the ten study sites, environmental variables were extracted from several sources of modelled or remotely-sensed data (fully described online in Supplementary Table S3). Chlorophyll‐*a* (Chl‐*a*, μg m^−3^) and suspended particulate inorganic matter (mg m^−3^) were extracted from SeaWiFS, MODIS and MERIS remotely-sensed products. Daily concentrations at 1 km^2^ resolution were provided as a merge of multiple-satellite data^71^ using an algorithm that has been validated against in-situ coastal observations for the English Channel and Bay of Biscay^72^. Hourly records of air and seawater temperatures, and salinity were obtained respectively from Météo France ARPEGE and CMEMS products and were averaged over the 30-day period preceding survey dates. The performance of air and seawater temperature products was tested by comparing their values with temperatures recorded in-situ over a period of 22 months^73^: three thermal sensors were deployed in the intertidal area of each of the ten sampled sites, however it was only possible to retrieve data from eight of these locations in summer and seven in winter (see Supplementary Fig. S2 online). Number and mean event duration of heatwaves and cold spells (as defined by) for both air and seawater were calculated using the “heatwaveR” package^74^. Cold spells and heatwaves were defined as a period of five or more days with temperatures warmer than the 90th percentile, or colder than the 10th percentile respectively^74,75^. Given our short time series, HW and CS detection was based on a historical baseline period starting from 2012^76^. Tidal amplitude data were obtained at the Atlantic scale (1/12°)^77^. A modified version of a wave exposure index was also calculated, where the hourly square of the wind speed (extracted from ARPEGE outputs) was multiplied by the wave fetch in the direction of the wind^78^. Wave fetch was calculated for each sampling location for all 360° using coastline polygon data from NOAA^79^ and the “fetchR” package^80^. The maximum distance for fetch segments was set to 300 km, and fetch was then standardised between 0 and 1 (with 0 representing a completely closed and 1 a completely open point), by dividing by the maximum fetch possible (360°/300 km). For each data layer, values within a 9 km buffer zone around the sampled site were averaged to get an accurate point estimate. All twelve variables (air and seawater temperature, air and seawater cold spells and heatwaves, salinity, current velocity, tidal amplitude, suspended particulate inorganic matter, chlorophyll and wave exposure) were then integrated (min, mean, max and 5, 25, 50, 75 and 95 quantiles) over the 30-day period preceding sampling dates (see Supplementary Table S3 online). This time interval was selected to be sufficient to capture the parental environment and allow the alteration of offspring phenotype^81^.

### Biochemical metrics

Five biochemical indicators of *S. alveolata*’s physiological condition were selected based on two previous studies on the same species^39,82^. Only individuals collected during winter 2018 were used, when the greatest proportion of ripe individuals are expected in anticipation of the spring phytoplankton bloom and the species main reproductive peak^68,83^. The polar lipid fatty acid composition of an organism reflects its physiological adaptation to its environment^84,85^. The long-chain polyunsaturated fatty acids 20:4n-6 (arachidonic acid, AA), 20:5n-3 (eicosapentaenoic acid, EPA) and 22:6n-3 (docosahexaenoic acid, DHA) are all considered essential for the survival, growth and reproduction of marine organisms^22^. Citrate synthase (CS), an enzyme involved in mitochondrial activity, is an indicator of aerobic metabolic rate and overall physiological condition^21^. Superoxide dismutase (SOD) is an anti-oxidant enzyme involved in the prevention of tissue damage from oxidative stress in marine invertebrates^86^. In order to have sufficient organic material for lipid analysis, all 60 frozen individuals were pooled into twelve batches of five individuals of similar size, as determined by their opercular crown diameter. Worm tissues were ground in liquid nitrogen with an MM400homogenizer (Retsch, Eragny, France). The resulting worm powder aliquots (100 mg) were homogenized in 2 ml chloroform–methanol (2:1, v/v,^87^), then sonicated and stored at − 20 °C. Neutral and polar lipid fatty acids, together with CS and SOD activity, were analysed following the protocol detailed in^82^.

### Reproductive trait metrics

The Flow Cytometer and Microscrope (FlowCAM^®^) system (Fluid Imaging Technologies, Scarborough, ME, USA) counts and analyses all individual particles in a fluid using high-speed digital imaging^88^. Observations were made using a Benchtop B3 Series FlowCAM with a 4× objective lens and a 300 µm-thick flow cell (FC 300). Worms were removed from the Falcon tubes and had their opercular crown diameter recorded. The 10 mL of spawning solution was vortexed before 0. 5 mL were sampled to run through the FlowCAM. In order to prevent clogging of the flow cell, samples were pre-filtered through a 200 µm gauze placed on top of the sample funnel. It is important to note that unfertilised *Sabellaria* spp. eggs produce a vitelline membrane immediately after contact with seawater^89^. We therefore define ‘total egg diameter’ as the diameter of the egg including the ovicell and vitelline membrane. When the number of particles per image exceeded 100, the sample was diluted by half with Milli-Q water and three diluted sub-samples were then dispensed. The instrument was run in auto image-mode, capturing all particles in the range of 15–200 μm. Based on this analysis, a threshold of at least 50 measured ovocytes per 0.5 mL was selected to retain samples for analysis, thus modulating the number of workable ripe individuals sampled to 2–34 individuals per site (see Supplementary Table S2 online). A preliminary Principal Component Analysis (PCA) of the 40 measurements taken by the software package VisualSpreadSheet^®^ version 4.3.55 showed that the following three reproductive traits captured 62–64.9% of the variation in over 440,000 ovocytes: (a) total egg diameter [µm], (b) best circle fit (deviation of the particle edge from a perfect circle, normalised to the range [0–1]) and (c) symmetry (real [0–1]) (see Supplementary Fig. S3 online).

### Statistical analyses

Individual clutch size is expressed as relative fecundity, i.e. the total number of eggs (calculated as the particle concentration in the 10 mL of spawning solution) standardised by opercular crown diameter. Any eggs with a diameter > 120 µm were excluded from analyses as they were assumed to be sample contamination from other species. One individual sampled from La Fontaine aux Bretons in winter 2018, out of 371 individuals in total, presenting an extremely high relative fecundity (> 400,000 eggs mm^−1^) was also removed from analyses so as not to distort multivariate and regression results, as values per site averaged between 4000 and 40,000 eggs mm^−1^ (Fig. 1).

### Latitudinal structures in reproductive and biochemical traits

Linear (ramped poleward/equator ward) or quadratic relationships (positive = abundant centre; negative = abundant edge) between the among-female mean reproductive and biochemical variables and latitude were tested using linear Model I regressions, with first and second-degree polynomials of latitude as explanatory variables (Fig. 1).

### Relationship between reproductive and biochemical traits

Common spatial structures between among-female mean biochemical (X) and reproductive traits (Y) of each site were searched through a co-inertia analysis^90^, where a PCA was computed on each dataset (Fig. 2). The RV coefficient^91^, a multivariate generalisation of the squared Pearson correlation^92^, was used to quantify the relationship between these two sets of descriptors. Ranging between 0 (independent) and 1 (homothetic), it measures the closeness between the two sets of points derived from separate ordinations of X and Y^93^.

### Drivers of variation in reproductive traits

The individual and combined contribution of local environmental conditions, biochemical variables and latitude in explaining within-clutch variation in total egg diameter and relative fecundity were quantified through a redundancy analysis (RDA) variance partitioning method^92,94^ applied to samples collected during winter 2018 (Fig. 3). Prior to the analysis, collinear environmental predictors were removed using a variance inflation factor threshold of 10^95^. A stepwise selection of the remaining variables was performed based on adjusted R^2^, with p-values for adding and dropping variables of 0.05 and 0.1, respectively. These explained fractions were put in perspective with the amount of within- *vs*. among-site variation found at each sampling season (see Supplementary Fig. S1 online). As different numbers of workable ripe individuals were sampled at each site, we used a re-sampling procedure to balance the dataset when quantifying the amount of within- vs. among-site variation. We excluded measures taken at Buarcos in summer as there were only two workable individuals. We used the lowest number of individuals measured within the remaining different sites to subsample the original data set, by randomly drawing 1000 data subsets with five individuals per site. Variance component models were performed on each subset to quantify, for each season, the variance in total egg diameter and relative fecundity across two nested scales (within- and among-site variation) (Fig. S1)^96^.

We additionally performed a RDA to better understand the role of environmental drivers^97^. Analyses were based on data collected during both seasons (Fig. 4) to understand the variation and covariation of all reproductive trait variables to environmental conditions during winter and summer. We included the within-clutch mean and standard deviation of the total egg diameter, best circle fit, symmetry and among-female mean relative fecundity as response variables, which we centered and standardised to unit variance, together with latitude (both linear and quadratic) and linear environmental predictors selected through the step-wise procedure described above.

All analyses were carried out with R 3.5.1^98^ using the *vegan* package^99^.

## Supplementary Information

Supplementary Information.

## Data Availability

Data associated with this study are available from Figshare at: 10.6084/m9.figshare.c.5081834.
