# Supplementary material for: Environmental optima for an ecosystem engineer: a multidisciplinary trait-based approach
Source: Sci Rep. 2021 Nov 26;11:22986. doi: 10.1038/s41598-021-02351-7 (PMC8626476; doi:10.1038/s41598-021-02351-7)
Supplement: Supplementary file 1 — Supplementary Information. [file 41598_2021_2351_MOESM1_ESM.pdf]

Environmental optima for an engineer species: a multidisciplinary trait-based approach

Authors

Amelia Curd[1*], Aurélien Boyé[1], Céline Cordier[1], Fabrice Pernet[2], Louise B. Firth[3], Laura E. Bush[4], Andrew J. Davies[5], Fernando P. Lima[6], Claudia Meneghesso[6,7], Claudie Quéré[2], Rui Seabra[6], Mickaël Vasquez[1] and Stanislas F. Dubois[1]

[1] IFREMER, Centre de Bretagne, DYNECO LEBCO, 29280 Plouzané, France.

[2] IFREMER, Centre de Bretagne, LEMAR UMR 6539, 29280 Plouzané, France.

[3] School of Biological and Marine Sciences, University of Plymouth, Drake Circus, Plymouth, PL4 8AA, United Kingdom.

[4] FUGRO GB Marine Limited, Gait 8, Research Park South, Heriot-Watt University, Edinburgh EH14 4AP, United Kingdom.

[5] Department of Biological Sciences, University of Rhode Island, Kingston, RI 02881, USA.

[6] CIBIO-InBIO, Centro de Investigação em Biodiversidade e Recursos Geneticos, Universidade do Porto, Campus Agrário de Vairão, 4485-661, Vairão, Portugal.

[7] Departamento de Biologia, Faculdade de Ciências da Universidade do Porto, R. Campo Alegre, s/n, 4169-007 Porto, Portugal

* Corresponding author. Tel: +33 298 224331

E-mail address: amelia.curd@ifremer.fr

Supplementary Figure S1

Boxplots of variance component analyses across two nested scales using a decomposition (varcomp function) of variance on restricted maximum likelihood (REML) method (lme function) on 1000 resampling of 5 individuals per site for a) total egg diameter in Summer 2017 b) total egg diameter in Winter 2018 c) relative fecundity in Summer 2017 and d) relative fecundity in Winter 2018. See Messier et al. 2010 for more information).

a) Total egg diameter, Summer 2017, 1000 resampling, 60% site, 40% intra-site

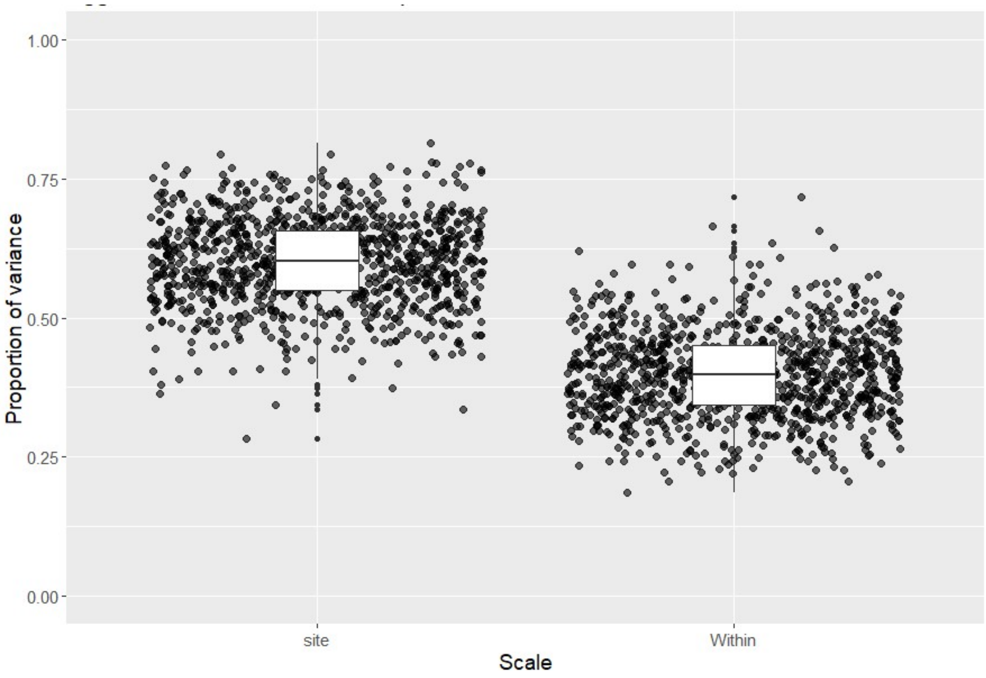

b) Total egg diameter, Winter 2018, 1000 resampling, 21% site, 79% intra-site

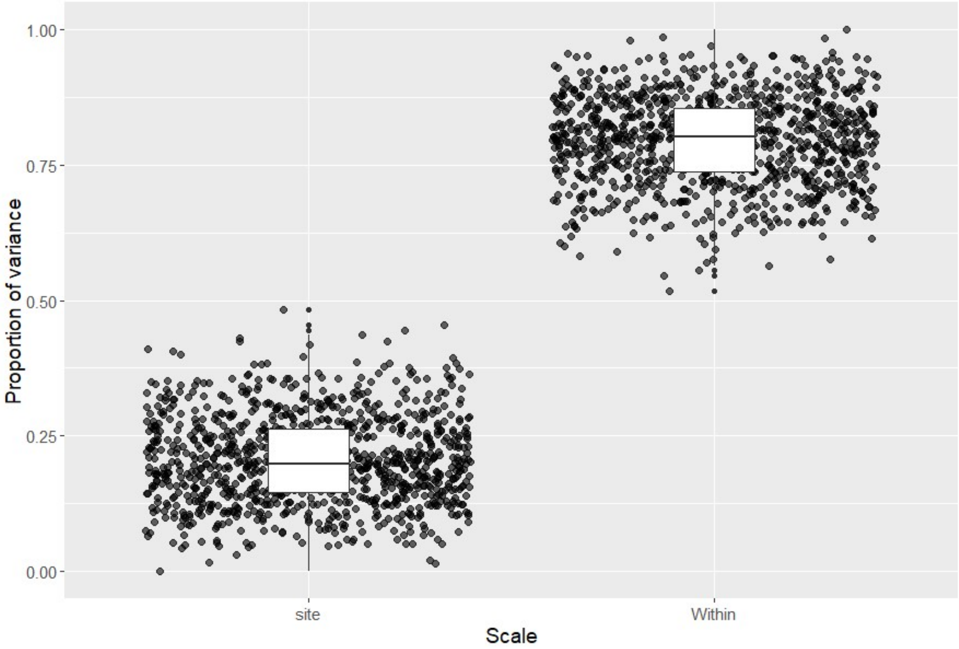

c) Relative Fecundity, Summer 2017, 1000 resampling, 22% site, 77% intra-site

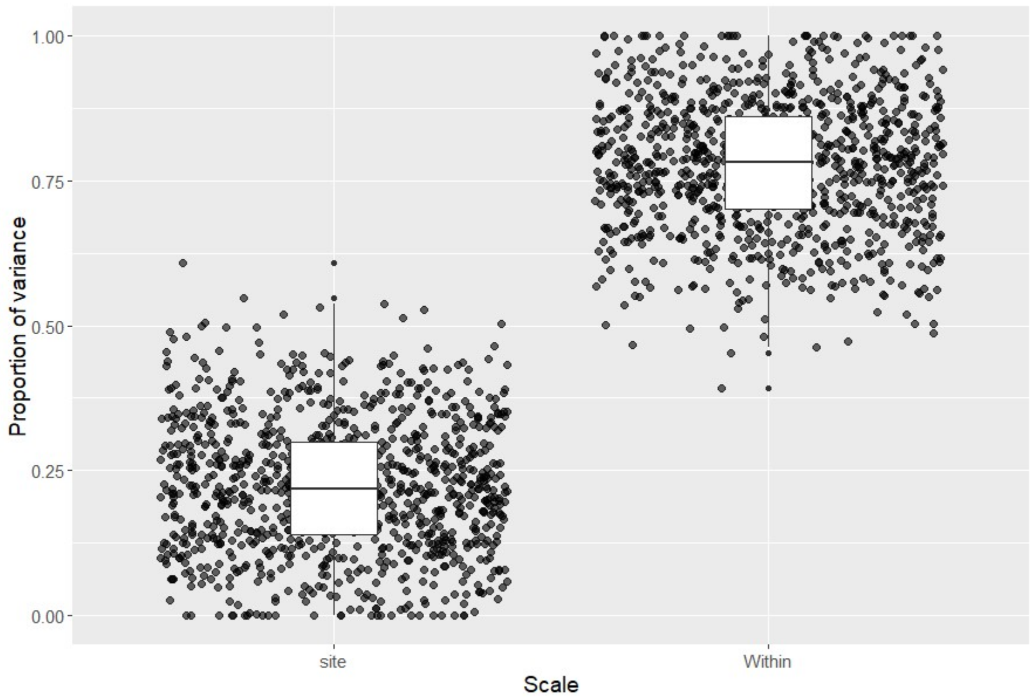

d) Relative Fecundity, Winter 2018, 1000 resampling, 41% site, 59% intra-site

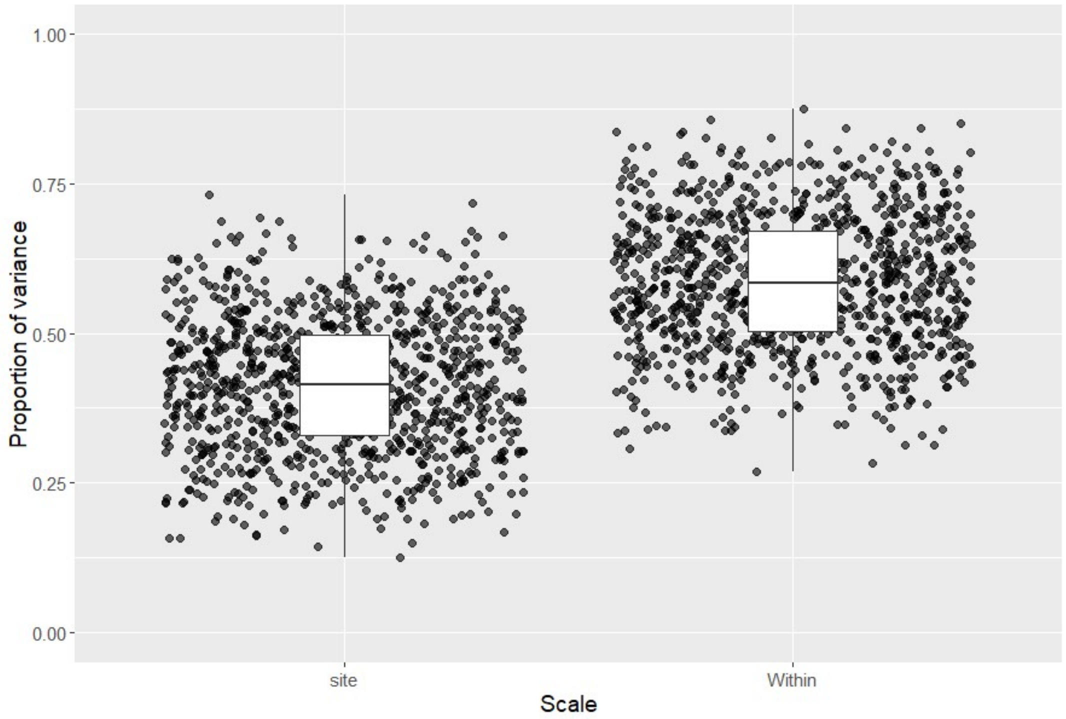

Supplementary Figure S2

Correlations between the *in-situ* temperature loggers and the environmental models over the 30-day period prior to sampling, for all project sampling dates (2016-2018 summer and winter, winter 2019). Please note that Maryport and Llanddulas loggers were not retrieved in summer 2017, and Maryport, Llanddulas and Douarnenez loggers were not retrieved in winter 2018. The red points and line, labelled "Emersion", represent the correlation between the ARPEGE model air temperature variable daily average and the logger readings at low-tide. The blue points and line, labelled "Immersion", represent the correlation between the CMEMS model seawater surface temperature daily average and the logger readings at high-tide. All correlations were significant at the p<0.001 level.

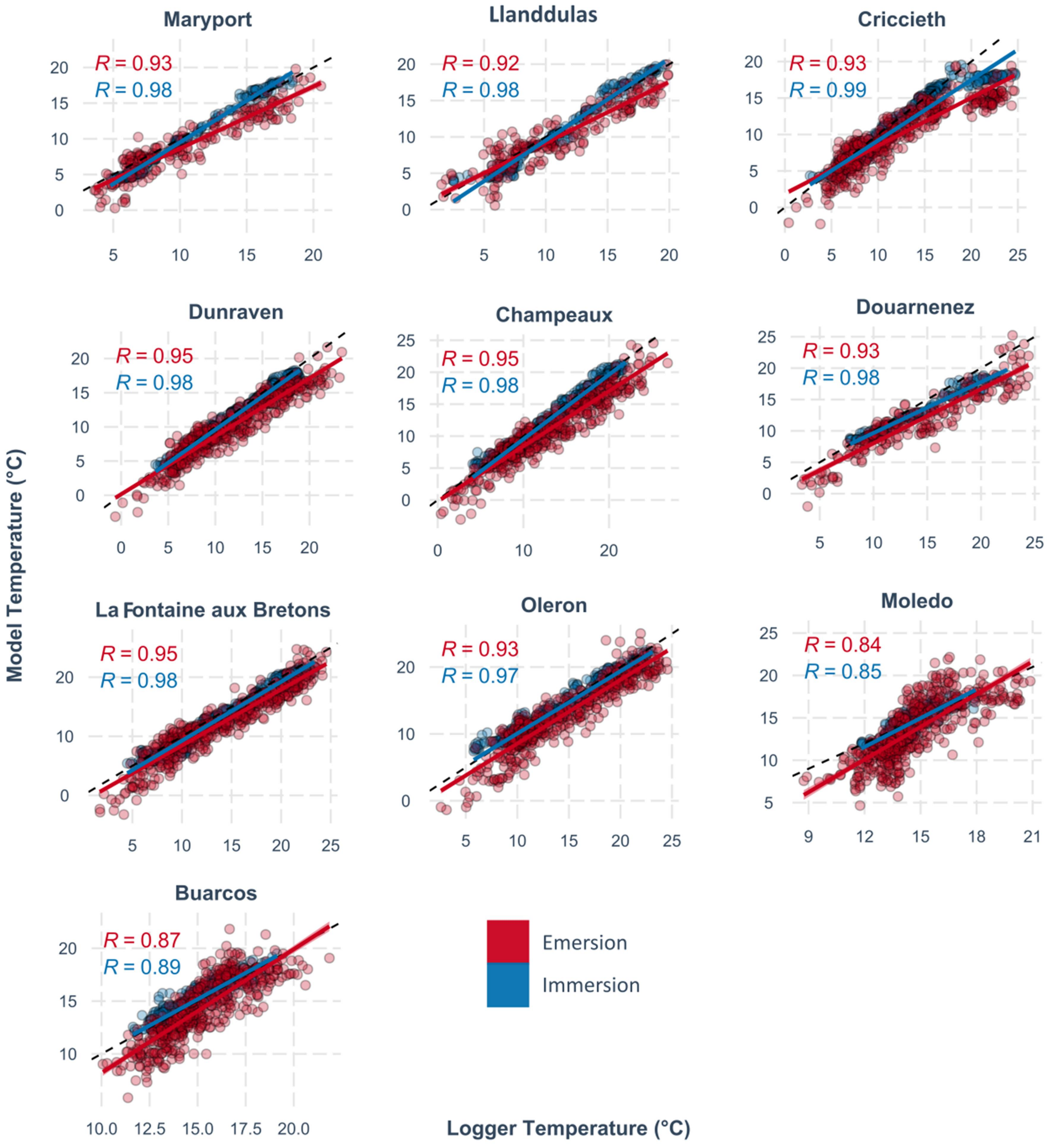

Supplementary Figure S3

Principal Component Analysis (PCA) plot of 440,000 *Sabellaria alveolata* eggs and FlowCAM parameters for both sampling periods.The first two dimensions of the PCA express 64.9% of the total dataset inertia in summer 2017, and 62% of the total dataset inertia in winter 2018. PCA calculated using the PCA function of the package factoextra (Kassambara and Mundt, 2019).

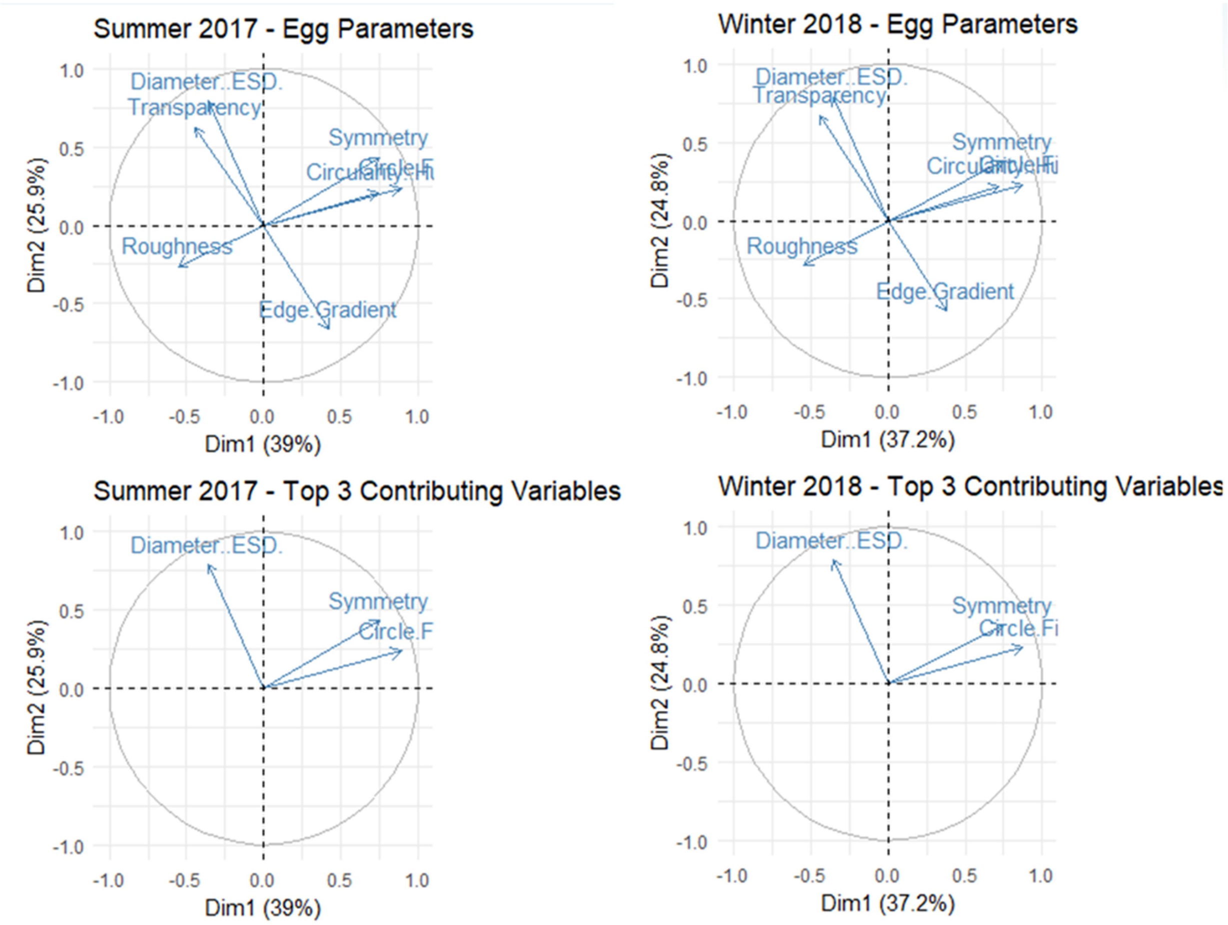

Supplementary Table S1. Key reproductive, environmental and biochemical variables and their relationship with either linear, or linear and quadratic, latitude. Graphical depictions of the regressions are presented. All variables are averaged at the site leve (9 sites in summer, 10 sites in winter). Regression equations are given (lat1 = linear latitude; lat2= second degree polynomial of latitude) as well as p-values and adjusted R^2 . Significant regression equations, when $p \leq 0.05$, are in bold. *** = $p \leq 0.001$, ** = $p \leq 0.01$, * $p \leq 0.05$. All fitted models were validated by checking that residuals were independent and normally distributed with mean zero and a constant variance.

	**Summer 2017**		**Winter 2018**	
**58° N**	**Linear**	**Quadratic**	**Linear**	**Quadratic**
	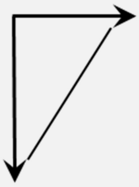	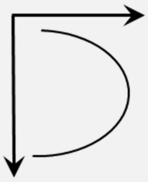	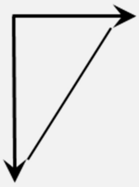	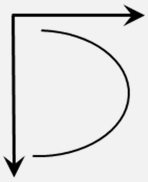
**40° N**	**~lat1**	**~lat1+lat2**	**~lat1**	**~lat1+lat2**
**Response variables**	(n=9)		(n= 10)	
Total egg diameter *μm*	p=0.18<br>Adj. R^2=0.14<br>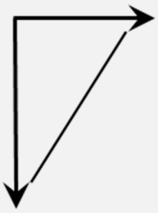<br>S17_ESDˆ= 78.43+14.29 (lat1)	p=0.06<br>Adj. R^2=0.60<br>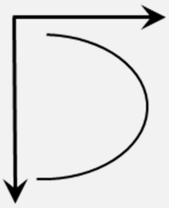<br>S17_ESDˆ=77.31 +13.25(lat1)+19.66 (lat2)	**p=0.03***<br>**Adj. R^2=0.38**<br>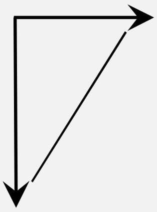<br>**W18_ESDˆ= 85.37−10.85 (lat1)**	p=0.12<br>Adj. R^2=0.29<br>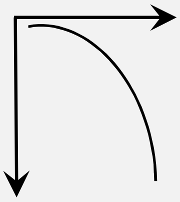<br>W18_ESDˆ=85.42 −10.82(lat1)−0.98 (lat2)
Relative fecundity *nb eggs.mm^-1*	p=0.15<br>Adj. R^2=-0.16<br>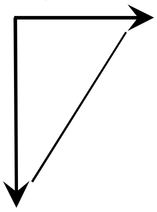<br>S17_rel_fecundˆ =21720.1+40813.86(lat1)	p=0.17<br>Adj. R^2=0.26<br>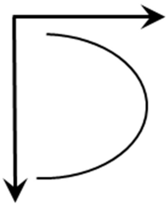<br>S17_rel_fecundˆ= 23861.76+42797.01(lat1)−37577.84 (lat2)	p=0.58<br>Adj. R^2=-0.08<br>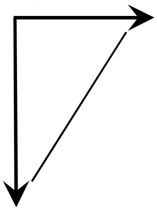<br>W18_rel_fecundˆ =10551.81−9962.52(lat1)	p=0.09<br>Adj. R^2=-0.36<br>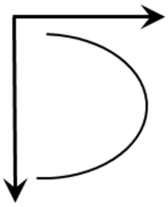<br>W18_rel_fecundˆ= 12643.29−8796.25(lat1)−39056.23 (lat2)
Circle Fit *[0-1]*	p=0.28<br>Adj. R^2=0.05<br>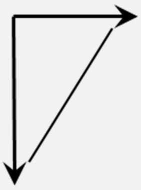	p=0.09<br>Adj. R^2=0.38<br>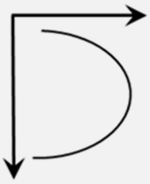	**p=0.05***<br>**Adj. R^2=0.31**<br>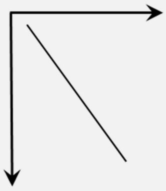	p=0.09<br>Adj. R^2=0.34<br>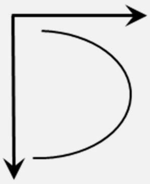

	S17_circleˆ =0.91+0.04(lat1)	S17_circleˆ=0.92 +0.05(lat1)−0.08 (lat2)	**W18_circleˆ =0.91−0.05(lat1)**	W18_circleˆ =0.91−0.04(lat 1)−0.03(lat2)
Symmetry *[0-1]*	p=0.41 Adj. R^2=-0.03 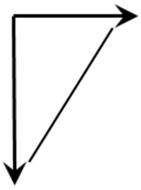 S17_symmetryˆ =0.92+0.04(lat1)	p=0.21 Adj. R^2=0.21 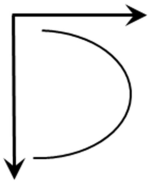 S17_symmetryˆ=0. 92+0.04(lat1)‐0.08(lat2)	p=0.09 Adj. R^2=0.23 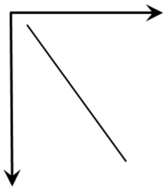 W18_symmetryˆ =0.93−0.04(lat1)	**p=0.05 Adj. R^2=0.44** 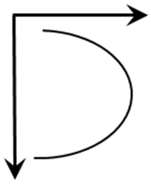 W18_symmetry =0.93−0.04(lat 1)−0.04(lat2)
**Env. variables**	**(n=9)**		**(n=10)**	
Mean Air Temperature *Degrees Celsius*	**p=0.02** Adj. R^2=0.50** 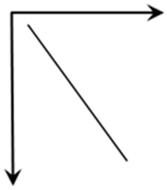 **air_mean_S17ˆ =17.11−14.81 (lat1)**	**p=0.01*** Adj. R^2=0.71** 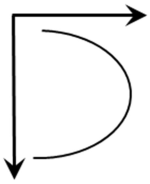 **air_mean_S17ˆ =17.72−14.25 (lat1)−10.6(lat2)**	**p=0.02* Adj. R^2=0.46** 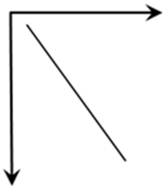 **air_mean_W18ˆ= 6.68−18.05(lat1)**	**p=0.004** Adj. R^2=0.74** 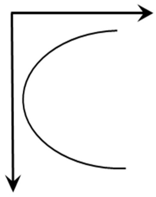 **air_mean_ W18^=5.87 −18.5(lat1) +15.23(lat2)**
Air Temperature SD *Degrees Celsius*	p=0.46 Adj. R^2=-0.05 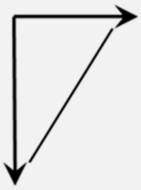 air_sd_S17ˆ =2.1+1.98(lat1)	**p=0.02* Adj. R^2=0.65** 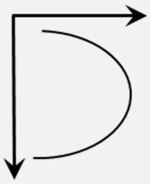 **air_sd_S17ˆ=2.47+ 2.33(lat1)−6.51 (lat2)**	p=0.11 Adj. R^2=0.20 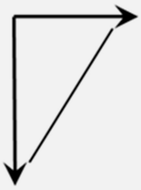 air_sd_W18ˆ =2.78+4.02(lat1)	**p=0.03* Adj. R^2=0.52** 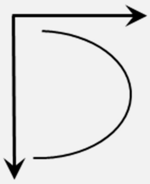 air_sd_W18ˆ =3.05+4.17(lat 1)−5.12(lat2)
Mean Seawater Temperature *Degrees Celsius*	p=0.50 Adj. R^2=-0.07 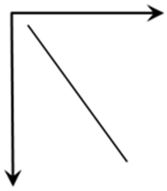 swtemp_mean_ S17ˆ=18.38+4.0 8(lat1)	p=0.09 Adj. R^2=0.38 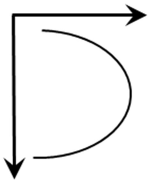 swtemp_mean_S17 ˆ=19.08+4.73(lat1)‐12.35(lat2)	**p<0.001*** Adj. R^2=0.94** 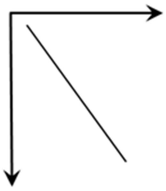 **swtemp_mean_ W18ˆ=8.12‐26.5 5 (lat1)**	**p<0.001*** Adj. R^2=0.93** 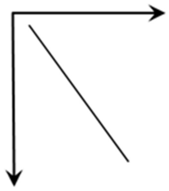 **swtemp_mean_ W18ˆ=8.09−26. 57(lat1)+0.43(la t2)**
Seawater Temperature SD *Degrees Celsius*	p=0.11 Adj. R^2=-0.23	p=0.25 Adj. R^2=0.16	p=0.08 Adj. R^2=0.26	**p=0.04* Adj. R^2=0.51**

	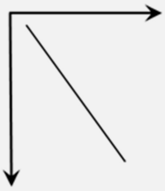<br>swtemp_sd_S17ˆ=0.76−1.65(lat1)	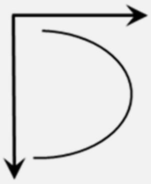<br>swtemp_sd_S17ˆ=0.72−1.69(lat1)+0.72(lat2)	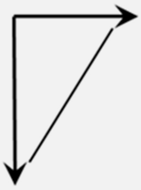<br>swtemp_sd_W18ˆ=0.53+1.28(lat1)	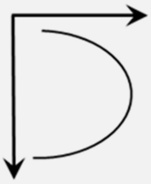<br>**swtemp_sd_W18ˆ=0.6+1.32(lat1)−1.34(lat2)**
Mean Chlorophyll *a*<br>*µg.m-3*	**p=0.01****<br>**Adj. R^2=0.57***<br>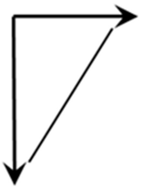<br>**chla_mean_S17ˆ=2.64+11.1(lat1)**	**p=0.003****<br>**Adj. R^2=0.81**<br>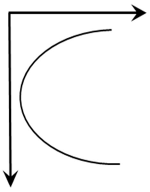<br>**chla_mean_S17ˆ=2.19+10.69(lat1)+7.87(lat2)**	p=0.32<br>Adj. R^2=0.01<br>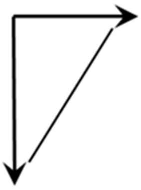<br>chla_mean_W18ˆ=2.06+3.44(lat1)	p=0.35<br>Adj. R^2=0.05<br>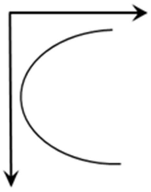<br>chla_mean_W18ˆ=1.83+3.32(lat1)+4.18(lat2)
Chlorophyll *a* SD<br>*µg.m-3*	p=0.22<br>Adj. R^2=0.09<br>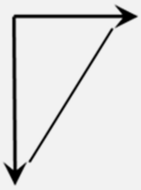<br>chla_sd_S17ˆ=0.77+1.8(lat1)	p=0.24<br>Adj. R^2=0.18<br>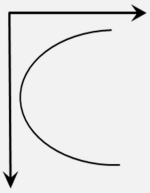<br>chla_sd_S17ˆ=0.66+1.7(lat1)+1.88(lat2)	p=0.58<br>Adj. R^2=-0.08<br>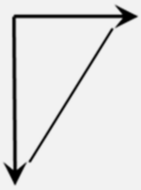<br>chla_sd_W18ˆ=0.55+0.83(lat1)	p=0.84<br>Adj. R^2=-0.22<br>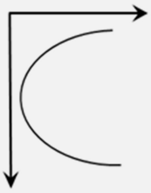<br>chla_sd_W18ˆ=0.53+0.82(lat1)+0.4(lat2)
Mean Salinity<br>*psu*	p=0.60<br>Adj. R^2=-0.10<br>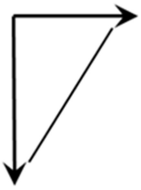<br>salinity_mean_S17ˆ=32.41+2.7(lat1)	p=0.78<br>Adj. R^2=-0.23<br>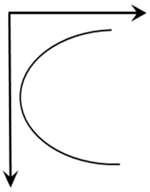<br>salinity_mean_S17ˆ=32.25+2.55(lat1)+2.96(lat2)	p=0.25<br>Adj. R^2=0.05<br>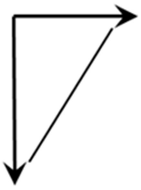<br>salinity_mean_W18ˆ=31.29+8.15(lat1)	p=0.44<br>Adj. R^2=-0.02<br>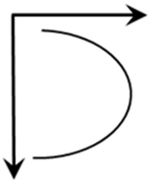<br>salinity_mean_W18ˆ=31.58+8.3(lat1)−5.29(lat2)
Mean Suspended Particulate Inorganic Matter<br>*mg.m-3*	p=0.37<br>Adj. R^2=-0.01<br>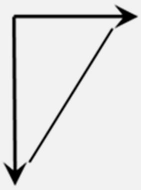<br>spim_mean_S17ˆ=5.42+15.29(lat1)	p=0.50<br>Adj. R^2=-0.06<br>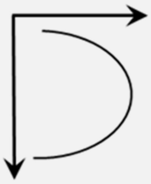<br>spim_mean_S17ˆ=6.3+16.11(lat1)−15.52(lat2)	p=0.66<br>Adj. R^2=-0.10<br>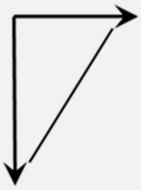<br>spim_mean_W18ˆ=22.85+29.74(lat1)	p=0.29<br>Adj. R^2=0.10<br>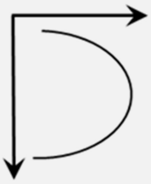<br>spim_mean_W18ˆ=28.93+33.13(lat1)−113.5(lat2)

Wave exposure<br>*(m.s-1)²*	p=0.31<br>Adj. R^2=0.03<br>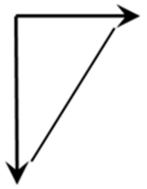<br>wave_exp_mean_S17ˆ=4.4+11.83(lat1)	p=0.51<br>Adj. R^2=-0.07<br>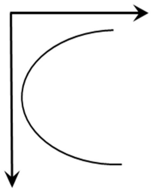<br>wave_exp_mean_S17ˆ=3.94+11.4(lat1)+8.2(lat2)	p=0.09<br>Adj. R^2=0.24<br>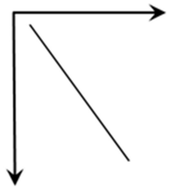<br>wave_exp_mean_W18ˆ=13.8−52.91(lat1)	**p=0.04***<br>**Adj. R^2=0.49**<br>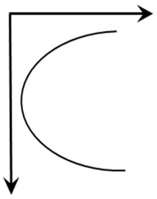<br>**wave_exp_mean_W18ˆ=10.75−54.61(lat1)+56.96(lat2)**
**Biochemical variables (n=10)**				
CS<br>*mU.mg⁻¹*			p=0.34<br>Adj.R^2=-0.01<br>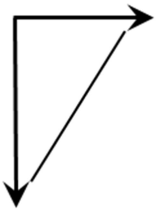<br>CSˆ=16.73+13.64(lat1)	**p=0.03***<br>**Adj. R^2=0.51**<br>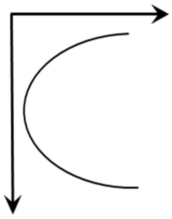<br>**CSˆ=14.96+12.65(lat1)+32.93(lat2)**
SOD<br>*U.mg⁻¹*			**p=0.05***<br>**Adj. R^2=0.31**<br>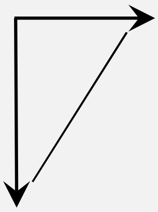<br>**SODˆ=3.68+4.42(lat1)**	**p=0.05***<br>**Adj. R^2=0.44**<br>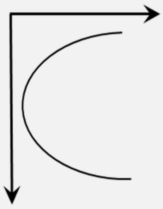<br>**SODˆ=3.49+4.32(lat1)+3.51(lat2)**
Polar Lipid Arachidonic Acid<br>*% of total phospholipids*			p=0.30<br>Adj. R^2=0.03<br>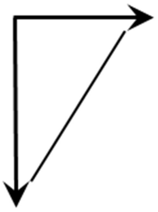<br>PL_AAˆ=1.79+1.52(lat1)	p=0.47<br>Adj. R^2=-0.04<br>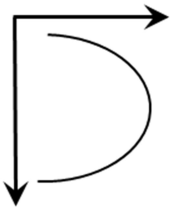<br>PL_20_4n.6ˆ=1.86+1.55(lat1)−1.17(lat2)
Polar Lipid EPA<br>*% of total phospholipids*			p=0.77<br>Adj.R^2=-0.11	p=0.73<br>Adj.R^2=-0.17

	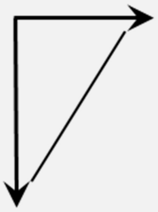<br>PL_EPA=21.67<br>+ 1.01(lat1)	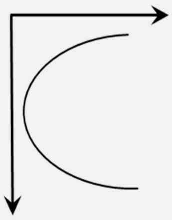<br>PL_20_5n.3ˆ=21.<br>83+1.11(lat1)<br>−3.11(lat2)
Polar<br>Lipid<br>DHA<br>*% of total phospholipids*	p=0.93<br>Adj.R^2=-0.12<br>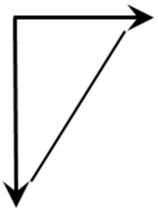<br>PL_DHAˆ=5.66<br>+0.45(lat1)	**p=0.05***<br>**Adj. R^2=0.43**<br>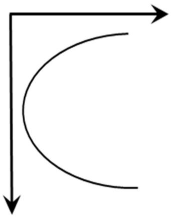<br>**PL_DHA^=5.02**<br>**+0.1(lat1)**<br>**+11.92(lat2)**

Supplementary Table S2. Field locations and sampling dates. Numbers in italic represent the number of viable individuals retained after FlowCAM quality controls (>50 measurable eggs per 0.5mL subsample).

Site name	Site abbreviations	GPS coordinates (decimal degrees)	Summer 2017 Sampling dates & No. of viable ind.	Winter 2018 Sampling dates & No. of viable ind.
Maryport	MAR	54.718869<br>-3.507036	21/08/2017<br>*24*	17/03/2018<br>*6*
Llanddulas	LLA	53.294723<br>-3.634655	21/08/2017<br>*13*	17/03/2018<br>*5*
Criccieth	CRI	52.916712<br>-4.230685	21/08/2017<br>*28*	17/03/2018<br>*9*
Dunraven	DUN	51.444295<br>-3.608907	11/06/2017<br>-	17/03/2018<br>*5*
Champeaux	CHA	48.732365<br>-1.552953	25/07/2017<br>*28*	01/03/2018<br>*31*
Douarnenez- plage du Ris	RIS	48.098813<br>-4.294829	24/07/2017<br>*24*	04/03/2018<br>*31*
La Fontaine aux Bretons	LFB	47.09958<br>-2.072538	22/07/2017<br>*34*	03/03/2018<br>*29*
Oléron	OLE	45.970809<br>-1.393057	21/07/2017<br>*28*	02/03/2018<br>*28*
Moledo do Minho	MOL	41.8417722<br>-8.9	25/07/2017<br>*24*	29/03/2018<br>*16*
Buarcos	BUA	40.1787<br>-8.9068	23/07/2017<br>*2*	30/03/2018<br>*6*

Supplementary Table S3. Environmental variable sources, units and horizontal resolution. All data were calculated over the thirty day period prior to the sampling dates.

**Variable**	**Abbreviated variable name**	**Unit**	**Source**	**Horizontal resolution**
Air temperature	air	Degrees C	ARPEGE*	~10km
Cold spells – air	arp_cs_n_event	No. of events over the 30-day period	HeatwaveR	~10km
Heatwaves – air	arp_hw_n_event	No. of events over the 30-day period	HeatwaveR	~10km
Seawater temperature	swtemp	Degrees Celsius	CMEMS†	~3km
Cold spells – water	cop_cs_n_event	No. of events over the 30-day period	HeatwaveR	~3km
Heat waves – water	cop_hw_n_event	No. of events over the 30-day period	HeatwaveR	~3km
Seawater Salinity	salinity	psu	CMEMS	~3km
Current Velocity	current_velocity	$m.s^{-1}$	CMEMS	~3km
Tidal amplitude	tide_amp	Meters	OTIS-OSU‡	~9km (1/12°)

Suspended Particulate Inorganic Matter	spim	$mg.m^{-3}$	OC5	~1km
Chlorophyll *a*	chla	$\mu g.m^{-3}$	OC5	~1km
Wave exposure index	wave_exposure	$(m.s^{-1})^2$	Adapted from Burrows et al. 2008	~10km

[*] = Météo-France European Centre for medium-range Weather Forecasts (ECMWF) atmospheric model (Déqué et al., 1994) [†] = EU Copernicus Marine Environment Monitoring Service (CMEMS) operational IBI (Iberian Biscay Irish) Ocean Physics Analysis and Forecast Product (IBI_ANALYSIS_FORECAST_PHYS_005_001) [‡]= Oregon State University Tidal Inversion Software - Regional Tidal Solution (Egbert & Erofeeva, 2010).

References

Déqué, M., Dreveton, C., Braun, A., Cariolle, D., 1994. The ARPEGE/IFS atmosphere model: a contribution to the French community climate modelling. Climate Dynamics 10, 249–266. https://doi.org/10.1007/BF00208992

Egbert, G.D., Erofeeva, S.Y., 2002. Efficient Inverse Modeling of Barotropic Ocean Tides. Journal of Atmospheric and Oceanic Technology 19, 183–204. https://doi.org/10.1175/1520-0426(2002)019<0183:EIMOBO>2.0.CO;2

Kassambara, A., Mundt, F., 2019 factoextra: Extract and Visualize the Results of Multivariate Data Analyses. R package version 1.0.6. https://CRAN.R-project.org/package=factoextra
